# Genomic Feature of a Rare Case of Mix Small-Cell and Large-Cell Neuroendocrine Lung Carcinoma: A Case Report

**DOI:** 10.3389/fonc.2021.794744

**Published:** 2022-01-18

**Authors:** Youcai Zhu, Feng Zhang, Dong Yu, Fang Wang, Manxiang Yin, Liangye Chen, Chun Xiao, Yueyan Huang, Feng Ding

**Affiliations:** ^1^ The Center for Thoracic Diseases, Zhejiang Rongjun Hospital, Jiaxing, China; ^2^ Department of Pathology, The Hospital of Marine Police Corps of the Chinese People’s Armed Police Force, Jiaxing, China; ^3^ Precision Medicine Center, Yangtze Delta Region Institute of Tsinghua University, Jiaxing, China; ^4^ Department of Pharmacy, Jiaxing University Medical College, Jiaxing, China; ^5^ Zhejiang Provincial Key Laboratory of Applied Enzymology, Yangtze Delta Region Institute of Tsinghua University, Jiaxing, China

**Keywords:** small cell lung cancer (SCLC), large-cell neuroendocrine lung carcinoma (LCNEC), genomic feature, FISH, case report, MET

## Abstract

**Background:**

Cases of both of small- (SCLC) and large-cell neuroendocrine lung carcinoma (LCNEC) were rarely reported. Although typical cases are morphologically distinct, the distinction between LCNEC and SCLC is still controversial, with some LCNECs showing close morphologies with SCLC. Here, we reported on a patient who had tumor with a mix of SCLC and LCNEC and uncovered these components’ histological and genomic features.

**Case Presentation:**

A 59-year-old man was diagnosed with lung cancer and had resection surgery in our hospital. The H&E and immunohistochemistry staining revealed that the tumor had 30%–35% LCNEC and 65%–70% SCLC cells. The whole-exome sequencing (WES) identified no potentially actionable alteration in the tumor sample but found five alterations all with allele frequency over 90%, including *TP53* p.R273H, *MYH8* p.Q1814K, *SLC17A6* p.W505L, *PTPN5* p.M40I, and *RB1* p.L267X. The genomic results supported that these two different components shared a similar dominant clonal origin. Furthermore, fluorescence *in situ* hybridization analysis revealed that the LCNECs have a higher copy number of MET than the SCLC component while without notable difference in the copy number of HER2 and TP53. Chemotherapy with pemetrexed and carboplatin was administrated for two cycles after the surgery. Although the chest CT showed remission in the lung, he was diagnosed with bone metastasis in 1 year later. Then, he received chemotherapy with etoposide and carboplatin but had severe side effect, leading to the discontinuation of the regime. Unfortunately, he returned to the local hospital with supportive care and died shortly after.

**Conclusion:**

Based on these observations, we proposed that LCNEC and SCLC components in this patient may have a common clonal origin with dual mutations in *TP53* and *RB1*, while the chromosome instability may cause multiple independent conversion that leads to LCNEC or SCLC morphologies.

## Introduction

Although only approximately 13% of all lung cancer cases are small-cell lung cancer (SCLC), it remains the sixth most common cause of cancer-related death worldwide due to early metastasis and rapid progression (1). Meanwhile, large-cell neuroendocrine lung carcinoma (LCNEC) represents roughly 3% of all lung cancer cases. According to the fourth edition of the World Health Organization classification of lung tumors, it is categorized as a neuroendocrine tumor with SCLC (2). SCLC and LCNEC are mainly distinguished by morphological features; however, the definitive distinction is still controversial (3, 4). Although typical cases are morphologically distinct, some LCNECs showed close morphologies with SCLC (3, 4). Recent molecular characterization shed new light on the classification of SCLC and LCNEC tumors. Here, we reported on a 59-year-old male patient who had tumor with a mix of SCLC and LCNEC and analyzed their histological and genomic features.

## Case Presentation

**Figure 1:**
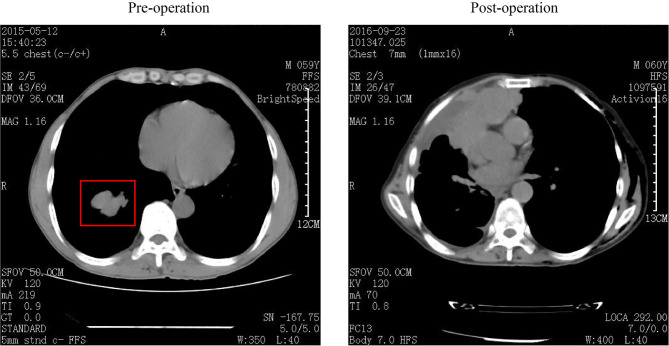
Computed tomography (CT) images of this patient. CT image was collected before (left) and after (right) surgery, and the tumor mass was labeled within the red box.

**Figure 2:**
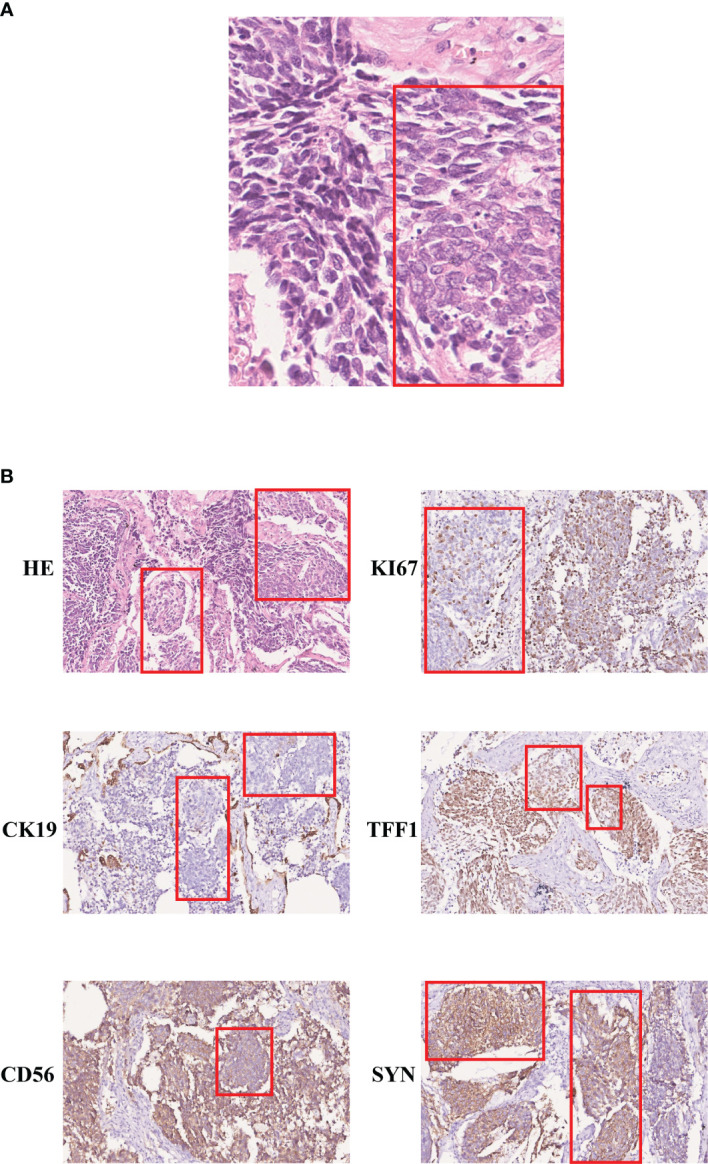
Immunohistochemistry staining of the SCLC and LCNEC regions. **(A)** H&E staining (400×); **(B)** immunohistochemistry staining of KI67, CK19, TFF1, CD56, and SYN (200×). The large-cell components were labeled within the red box.

A 59-year-old man was transferred to our hospital in May 2015, with a 4.8 × 3.5 cm nodule with clear boundaries in the right lower field revealed by the chest computed tomography (**Figure 1**). Then, surgery was performed with video-assisted thoracoscopic resection of the right lower lobe and lymph nodes. The pathological evaluation showed a 6.0 × 4.0 × 3.3 cm tumor mass, and by hematoxylin–eosin (H&E) staining and immunohistochemistry (IHC) staining, it was demonstrated that 30%–35% of the tumor cells were LCNEC, and the rest 65%–70% were SCLC (**Figure 2A**). Both the small- and large-cell components were positive for NCAM (CD56), synaptophysin (Syn), and thyroid transcription factor-1 (TFF1) but negative for cytokeratin 19 (CK19), which were indicative of neuroendocrine tumor. The Ki67 staining was positive for both the small- and the large-cell components, with the small cells having a high percentage of positive cells (67.5% versus 47.5%, **Figure 2B**).

**Figure 3:**
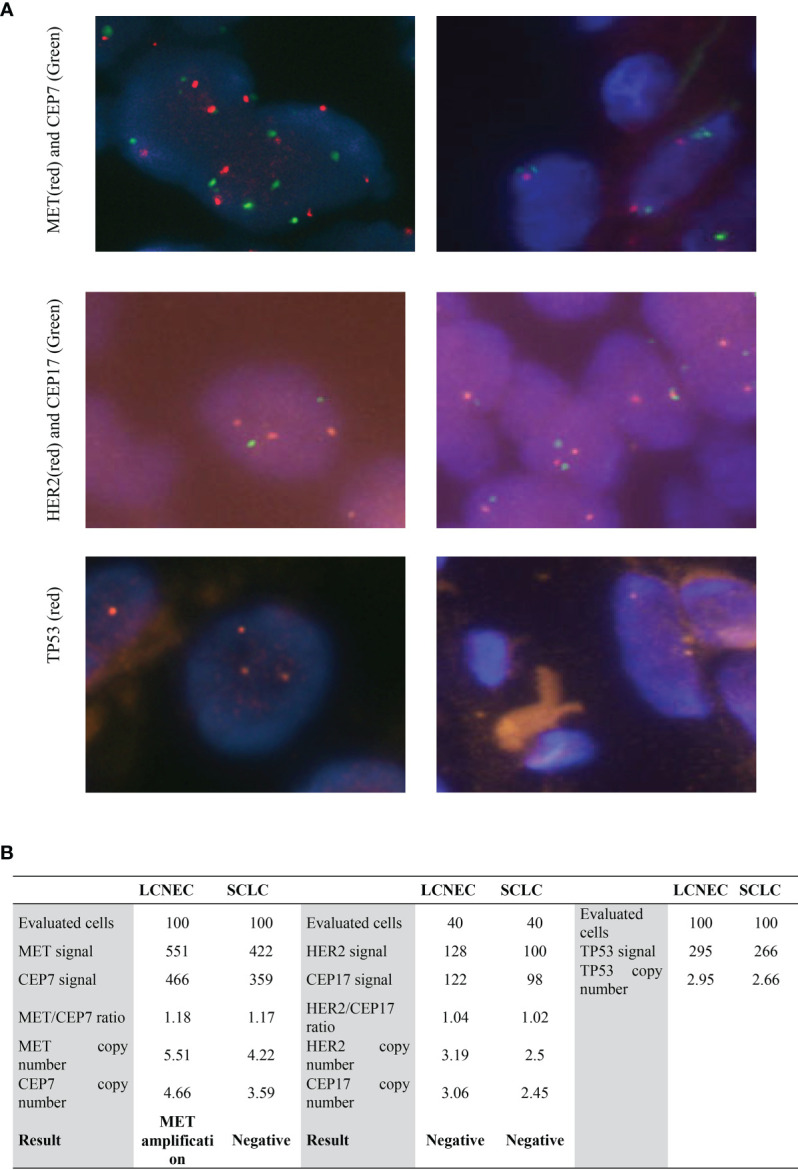
Results of MET, HER2, and TP53 FISH in large- and small-cell components. **(A)** FISH images of MET, HER2, and TP53 FISH in LCNEC (left) and SCLC components (right). The magnification was 1,000×. **(B)** Quantification of MET, HER2, and TP53 copy number in FISH. LCNEC, large-cell neuroendocrine lung carcinoma; SCLC, small-cell lung cancer.

In order to identify actionable genomic alterations to guide patient’s treatment, genetic testing of the whole tumor sample was performed. However, the whole-exome sequencing (WES) identified no actionable alteration in the tumor samples. WES data showed that, in addition to the high allele frequency (AF) of *TP53* R273H (AF, 98.9%), which is a well-studied pathogenic mutation, alterations with high allele frequency were found in *MYH8* (95.3%), *SCL17A6* (93.1%), *PTPN5* (92.1%), and *RB1* (90.0%) (**Table 1**), indicating that both the SCLC and the LCNEC components were of the same mutant genotype. The *RB1* c.799delC mutation was not reported in the ClinVar or COSMIC database, and as it resulted in a premature stop codon (p.L267X) that led to a non-functional protein, so it was classified as a novel pathogenic mutation. The *SCL17A6* p.W505L was also not presented in ClinVar database but had been identified previously in lung cancer as documented in the COSMIC database with a highly pathogenic FATHMM score of 0.99. The *MYH8* p.Q1814K and *PTPN5* p.M40I had not been reported in the COSMIC database, indicating that they are likely to be novel mutations. Furthermore, fluorescence *in situ* hybridization test (**Figure 3**) showed that *MET* was amplified in the large-cell components with an average copy number of 5.51, whereas for the small-cell component, the *MET* copy number was gained to 4.22 but did not reach the threshold of five copies per cell. To find out whether the cells were polyploid, HER2, CEP17, and TP53 were also tested, and three copies of HER2, CEP17, and TP53 (**Figure 3**) were detected in the large-cell component, but less than three copies of HER2, CEP17, and TP53 were detected in the small-cell component (**Supplemental Figure S1**). These results indicated that the large and small components of the tumor had different ploidy, which were also validated by the evaluation of the single nucleotide polymorphism (SNP) frequency generated by WES of the tumor and non-tumoral lymph node samples. Additional whole chromosome trisomy was found on Chr3, 21, and 22; regional trisomy was found on Chr5, 9, and 11, and loss of heterozygosity (LOH) was found on Chr11 and 13 (**Supplemental Figure S2**).

**Table 1:** High allele frequency mutations identified by WES in the tumor sample.

Chr	Gene	Freq	Mut/Wt	Transcript	cDNA	Protein	COSMIC	FATHMM	χ^2^ test
17	TP53	98.9%	117/1	NM_000546	c.G818A	p.R273H	10660	Pathogenic	4.8E−12
17	MYH8	95.3%	212/13	NM_002472	c.C5440A	p.Q1814K	None	Unknown	2.2E−15
11	SLC17A6	93.1%	58/2	NM_020346	c.G1514T	p.W505L	6132215	Pathogenic	6.6E−06
11	PTPN5	92.1%	76/3	NM_006906	c.G120T	p.M40I	None	Unknown	3.7E−07
13	RB1	90.0%	30/3	NM_000321	c.799delC	p.L267X	None	Pathogenic	8.8E−03

The χ^2^ test was done against an expected frequency of 70%.

Chemotherapy of 800 mg pemetrexed and 400 mg carboplatin was administrated for two cycles after the surgery. A year later, chest CT showed remission in the lung, but he was diagnosed with bone metastasis. Then, chemotherapy with 100 mg × 3 etoposide and 200 mg carboplatin was administrated. Unfortunately, the patient had severe side effect and did not continue with the regime; then, he returned to the local hospital with supportive care but died shortly after.

## Material and Methods

### Immunohistochemistry

Formalin-fixed, paraffin-embedded (FFPE) tissue blocks were cut into 4-μm sections, deparaffinized in xylene, and rehydrated in a graded series of ethanol. Antigen retrieval was performed using citric (CK19, CD56, synaptophysin) or Tris–EDTA buffer (TTF-1 and Ki67). Immunohistochemistry was performed using primary antibodies and ultrasensitive second antibody kit (PV-9000, Zsbio Inc., Beijing). The following primary antibody working solutions were used: CK19(ZM-0074), CD56 (ZM-0057), synaptophysin (Syn) (ZM-0246), TTF-1 (ZM-0250), and Ki67 (ZM-166).

### DNA Extraction From FFPE Tissue

The FFPE sections were deparaffinized in dewaxing agent (Wuxi Jiangyuan Industrial and Trade Co., Jiangsu, China) at 60°C for 1 min, washed with 100% ethanol at room temperature, and air dried for 10 min. Genomic DNA was isolated from the tumor and lymph node FFPE samples by using the Biomark FFPE Genomic DNA Kit (Beijing ACCB Biotech, Beijing, China) in accordance with the manufacturer’s instructions.

### Whole-Exome Sequencing and Data Analysis

DNA from FFPE sections of the tumor or lymph node were sequenced by Bionova (Beijing, China). Briefly, the DNA samples were fragmented and captured by IDT’s xGenExome Research Panel (Integrated DNA Technologies, San Diego, USA) and sequenced by using the Illumina HiSeq™4000 platform with 150 bp pair-end reads with a total coverage of 200×. The sequencing reads were aligned to the human reference genome hg19/GRCh37 using the Burrows–Wheeler Aligner tool, and the PCR duplicates were removed by using Picard v1.57 (http://picard.sourceforge.net/). GATK (https://software.broadinstitute.org/gatk/) were employed for variant calling. Variant annotation and interpretation were conducted through the use of ANNOVAR. Somatic mutations were defined as mutations found in the tumor tissue of the patient but not in the cancer-free lymph node.

## FISH

FFPE sections of the tumor and lymph node were pretreated with Vysis Paraffin Pretreatment IV (Abbott Molecular, IL) according to the manufacture’s instruction. Probe mixture for HER2, MET, and TP53/CEP17 (Abbott Molecular, IL) was added onto the hybridization area, then coverslipped and sealed with rubber cement. Slides were incubated in Termobrite (Abbott) at 73°C for 5 min (HER2, TP53/CEP17) or 73°C for 3 min (MET) for denaturation, and hybridized at 37°C overnight. The sections were washed by using Post-Hybridization Wash Buffer Kit (Abbott). After gently removing the rubber cement and coverslip, the slides were washed in Washing Buffer II (HER2, TP53/CEP17) at 72°C for 2 min or Washing Buffer II (MET) at 74°C for 2 min. Then, the slides were washed briefly in 70% EtOH, air-dried in darkness, and stained with 4′,6-diamidino-2-phenylindole (DAPI) counterstain and coverslipped. FISH results were examined with a BX43 fluorescence microscope (Olympus), and photographs were taken with a digital camera (CellSens) by using appropriate filters.

## Discussion

Although SCLC and LCNEC are distinguished by morphological features, the expression of the neuroendocrine markers such as CD56 and synaptophysin is indicative of a similar origin (3, 4). Recent molecular characterization showed that SCLC and LCNEC tumors had overlapping mutation profiles, which complicated their classification. In this study, the histological and genomic feature of a rare case of mix SCLC and LCNEC was analyzed. Although the tumor sample contained about a third of LCNEC cells, pathogenic alterations *TP53* p.R273H and *RB1* p.L267X were found at an AF of 98.9% and 90%, respectively, indicating that both the SCLC and LCNEC components harbored the pathogenic *TP53* and *RB1* alterations. The dual inactivation of *TP53* and *RB1* is a prominent feature for SCLC as reported by multiple independent studies (5–8). For LCNEC, genetic and gene expression analysis of 45 morphologically identified cases showed that 40% cases were SCLC-like as characterized by *TP53* and *RB1* co-mutation and gene expression profiles, and the rest 56% had the NSCLC-like profiles instead, lacking dual mutation in *TP53* and *RB1* (9). Since both LCNEC and SCLC are neuroendocrine tumors, in a study of 148 lung neuroendocrine tumors that included LCNEC, SCLC, and carcinoids, distinct mutational landscape was noticed for carcinoids and carcinomas, but LCNEC and SCLC showed similar mutational profiles except for the high prevalence of *RB1* mutation in SCLC, and *SMARCA2* mutation is found exclusively in LCNEC (10). A recent study on LCNEC, SCLC, and LC showed that *RUNX1*, *ERBB4*, *BRCA1*, and *EPHA3* distinctively mutated in LCNEC, although the mutation frequency was moderate, and consistent with a previous study, 4/14 of LCNEC cases showed dual inactivation mutation in *TP53* and *RB1* (11). The result of the current study is in line with these reports, which highlighted the similarity of a subset of LCNEC to SCLC. Yet, due to that the SCLC and LCNEC cases were of independent patients, it is hard to conclude whether the SCLC and LCNEC subset had the same oncogenesis path. The current case study offered a unique opportunity to study the origin of SCLC and LCNEC. First, the SCLC and LCNEC components were derived from the same patient, rendering them identical in genetic background and environmental influences. Second, the SCLC and LCNEC components did not originate from separate locations but were present as multiple intermingled nests. Third, in addition to *TP53* and *RB1*, high-frequency mutations in genes such as *MYH8* (95.3%), *SCL17A6* (93.1%), and *PTPN5* (92.1%), which located on different chromosomes, were also identified. This indicated that the similarity of genetic mutation in SCLC and LCNEC components are unlikely to be originated independently; a more likely scenario is that the SCLC and LCNEC components had the same origin of early oncogenesis, and they were derived from the same mutant clone that harbors these mutations.

If the SCLC and the LCNEC components originated from the same clone, why were they of different morphologies? To answer this question, the best study would be to isolate the SCLC and LCNEC components and perform mutation and gene expression analysis on them. The intermingled growth of the SCLC and LCNEC components, however, made the dissection technically difficult. FISH study at the single cell level allowed a preliminary evaluation of the genetic differences of the two components. We found that the LCNEC portion had slightly higher copy numbers in *MET*, which indicated that after the initial clonal growth, subsets of cells diverged. Although *MET* copy number was above the threshold as a biomarker for TKI treatment, the SCLC had a higher Ki67 levels than the LCNEC component.

For patients with mix pathological tumor components, the prognosis was usually poor. The heterogeneity *per se* may indicate a high level of genomic instability, which renders the tumor a higher chance to mutate and gain drug-resistant features. In addition, the subclones or the heterogeneous components may contain different signal transduction pathways, and the inhibition of one pathway may hinder the growth of a portion of cells but not the rest. The development of drugs that targets different subclones/components may be necessary for the effective control of tumor growth.

In summary, this study reports a rare case of mix SCLC and LCNEC. The molecular analysis indicated that the SCLC and LCNEC were derived from the same early clone that harbors *TP53* and *RB1* null mutations, and mutations in *MYH8*, *SCL17A6*, and *PTPN5*. We propose that LCNEC containing dual mutations in *TP53* and *RB1* can have a common clonal origin with SCLC, with the genomic instability that causes additional mutations for the diversion to LCNEC or SCLC.

## Data Availability Statement

The datasets presented in this study can be found in online repositories. The names of the repository/repositories and accession number(s) can be found in the article/**Supplementary Material**.

## Ethics Statement

The studies involving human participants were reviewed and approved by the Ethics Committee of Jiaxing University. The patients/participants provided their written informed consent to participate in this study.

## Author Contributions

YZ: manuscript writing. FZ: data collection and analysis. DY: data collection. FW: data collection. MY: data collection. LC: data collection. CX: data collection. YH: project development and data collection. FD: project development and manuscript writing. All authors contributed to the article and approved the submitted version.

## Conflict of Interest

The authors declare that the research was conducted in the absence of any commercial or financial relationships that could be construed as a potential conflict of interest.

## Publisher’s Note

All claims expressed in this article are solely those of the authors and do not necessarily represent those of their affiliated organizations, or those of the publisher, the editors and the reviewers. Any product that may be evaluated in this article, or claim that may be made by its manufacturer, is not guaranteed or endorsed by the publisher.
