# Supplementary material for: Genomic Feature of a Rare Case of Mix Small-Cell and Large-Cell Neuroendocrine Lung Carcinoma: A Case Report
Source: Front Oncol. 2022 Jan 18;11:794744. doi: 10.3389/fonc.2021.794744 (PMC8804208; doi:10.3389/fonc.2021.794744)

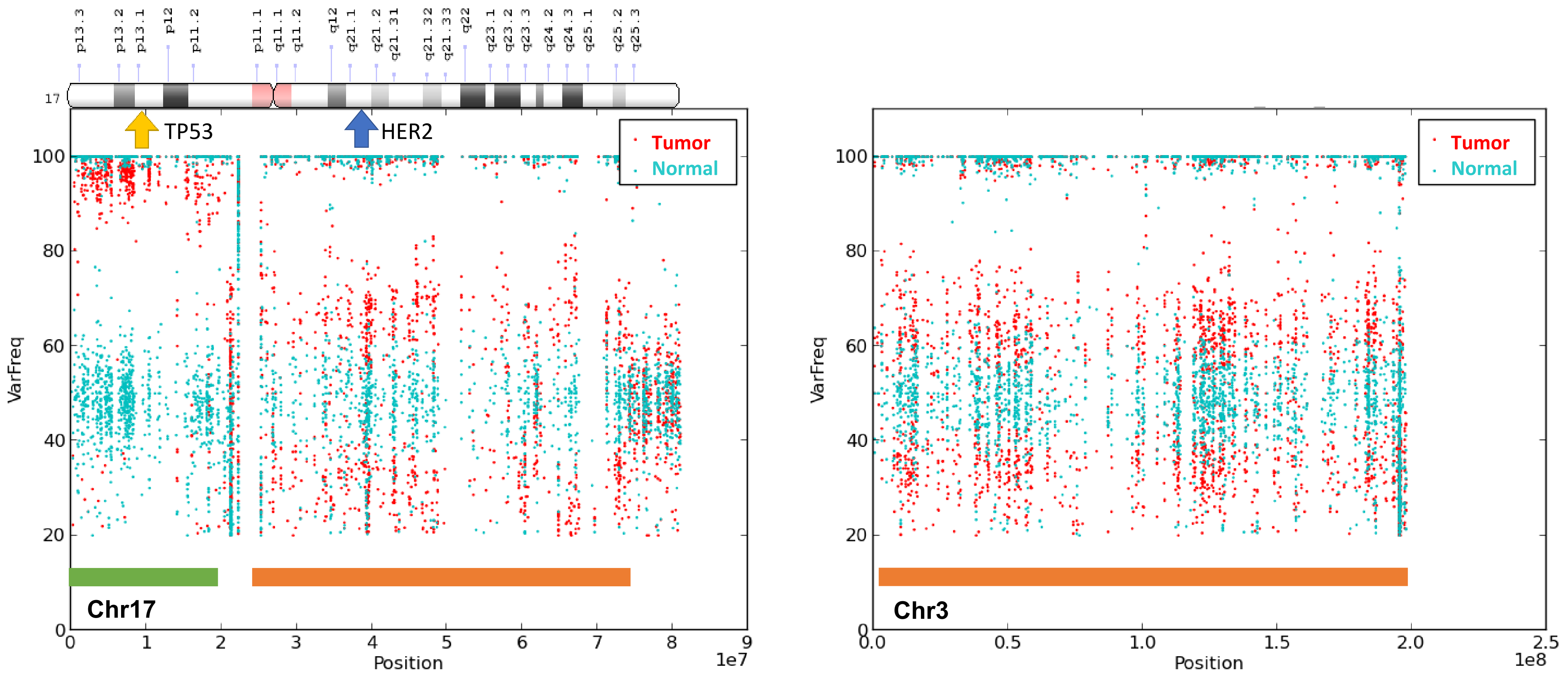

Supplemental figure 1: SNP frequencies of WES analysis of tumor and negative lymph node A. SNP distribution indicative of LOH of the Chr 17p arm and trisomy of the 17q arm. The approximal location of TP53 and HER2 were shown by yellow and blue arrows, respectively. B. SNP distribution indicative of trisomy of Chr 3 in tumor sample. Red dots, SNPs from the tumor; turquoise dots, SNPs from normal lymph node. Green bar, LOH region; orange bar, trisomic region.

Supplemental Figure 2.SNP frequencies of chromosomes that contained LOH or trisomic regions based on the WES analysis on tumor and negative lymph node. Red dots, SNPs from the tumor; turquoise dots, SNPs from normal lymph node. Green bar, LOH region; orange bar, trisomic region.

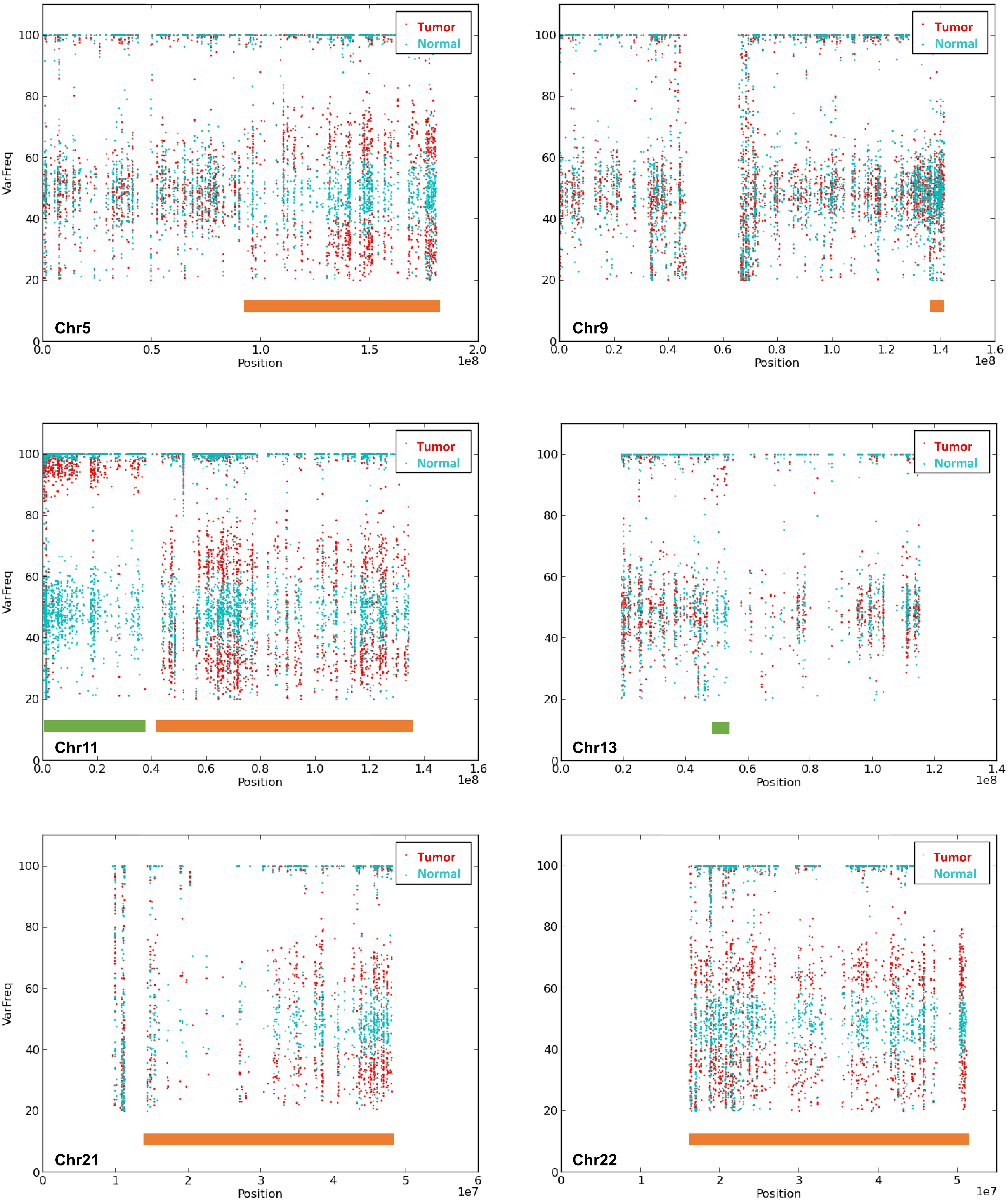

Supplement: Supplementary file 1 [file DataSheet_1.pdf]
